# Transition of the intestinal microbiota of cats with age

**DOI:** 10.1371/journal.pone.0181739

**Published:** 2017-08-16

**Authors:** Hiroaki Masuoka, Kouya Shimada, Tomoyo Kiyosue-Yasuda, Masaharu Kiyosue, Yukie Oishi, Seiji Kimura, Yuji Ohashi, Tomohiko Fujisawa, Kozue Hotta, Akio Yamada, Kazuhiro Hirayama

**Affiliations:** 1 Laboratory of Veterinary Public Health, Graduate School of Agricultural and Life Sciences, The University of Tokyo, Yayoi, Bunkyo-ku, Tokyo, Japan; 2 Nisshin Petfood Inc., Chiyoda-ku, Tokyo, Japan; 3 Laboratory of Food Hygiene, Department of Food and Technology, Nippon Veterinary and Life Science University, Kyonanchou, Musahino-shi, Tokyo, Japan; Virginia Polytechnic Institute and State University, UNITED STATES

## Abstract

The transition of intestinal microbiota with age has been well described in humans. However, the age-related changes in intestinal microbiota of cats have not been well studied. In the present study, we investigated the composition of intestinal microbiota of cats in 5 different age groups (pre-weanling, weanling, young, aged, senile) with a culture-based method. For lactobacilli and bifidobacteria, we also quantified with molecular-based method, real-time PCR. The results suggested that the composition of the feline intestinal microbiota changes with age, while the changes were different from those of humans and dogs. Bifidobacteria which are predominant in human intestine or lactobacilli which are predominant in dog intestine, did not appear to be important in cat intestines. Enterococci, instead, seem to be major lactic acid producing bacteria in cats. We also identified lactobacilli and bifidobacteria at the species level based on 16S rRNA gene sequences and found that the species composition of *Lactobacillus* also changed with age.

## Introduction

The cat is one of the most popular companion animals around the world and is playing important roles as a member of the family. Recent advances of veterinary practices have dramatically prolonged the life span of cats, making disorders associated with aging in the aged cat population more serious problem.

It is now well accepted that the intestinal microbiota has great impacts on health and disease of the host, and the impacts are both in beneficial and harmful ways. Therefore, maintaining the intestinal microbiota in good condition is important for good health of the host. It has been reported that the composition of the intestinal microbiota is influenced by various factors. Among the many factors that influence the composition of the intestinal microbiota, age is one of the most critical [1].

It has been reported that the composition of the human intestinal microbiota changes with age, and “aging of the intestinal microbiota” is thought to be somehow related to the health of the host [1]. For example, it was revealed that bifidobacteria, which are also often used as probiotics, decrease during the transition from middle age to old age, while the numbers of *Clostridium perfringens*, lactobacilli, enterococci and Enterobacteriaceae increase [1]. Recently, the authors have reported the transition of intestinal microbiota of dogs with age [2]. However, although the compositions of the intestinal microbiota of various animal species have been studied [1, 3, 4], transition of the intestinal microbiota of cats with age has not been well studied.

In this study, we analyzed the composition of the intestinal microbiota in cats of different age groups to elucidate the age-dependent transitions.

## Materials and methods

### Animals

In this study, we collected fresh fecal samples from 5 different age groups of cats, and each group contained 10 animals (Table 1). Animals in pre-weanling and weanling groups were 12 to 13 days old and 7 to 8 weeks old respectively. Young cats were 2 to 3 years old, and aged cats were 10 to 14 years old. Pre-weanling, weanling, young and aged cats were mixed breed bred and maintained at Kitayama Labes Co., Ltd (Nagano, Japan). Pre-weanling cats were breast-fed and housed in group. Weanling, young and aged cats were housed individually and fed CF6 diet (Oriental Yeast Co., Ltd, Tokyo, Japan). On the other hands, senile cats were 16 to 19 years old mixed breed except for one American shorthair. Two of them were maintained individually at Kitayama Labes Co., Ltd and fed CF6 diet. Eight senile cats were kept in owner household and fed commercial diet (JP-style dietetics kidney keep (Nisshin Petfood) for 1, Neko Genki series (Unicharm) for 2, Gin no spoon series (Unicharm) for 2, KalKan (Mars) for 2, Royal Canin pH control (Royal Canin) for 1). Owners provided written informed consent before collection of the samples. All cats were apparently healthy and had no symptom of illness except for a senile cat with urinary calculus and a senile cat with renal disease.

**Table 1:** Cats used in this study.

	n	age	
pre-weanling	10	12.6 ± 0.5	days
weanling	10	7.5 ± 0.5	weeks
young	10	2.5 ± 0.5	years
aged	10	11.4 ± 1.6	years
senile	10	17.5 ± 1.2	years

### Collection of fecal samples

Fresh fecal samples were collected immediately after defecation avoiding contamination from the environment and kept under anaerobic conditions with AnaeroPack^®^ Kenki (Mitsubishi Gas Chemical Company Inc., Tokyo, Japan). In the case of the pre-weanling group, kittens were provoked to defecate. Samples were refrigerated and transported to Laboratory of Veterinary Public Health, the University of Tokyo, the next day. Samples from the senile cats were aseptically collected immediately after defecation at their veterinary clinic and transferred to the laboratory in the same way.

### Bacteriological procedures

Bacteriological procedures were essentially the same as those described previously [1, 5]. Samples were weighed and introduced into an anaerobic chamber (85% N_2_, 5% CO_2_, and 10% H_2_), and 10-fold serial dilutions were prepared with prereduced trypticase soy broth without dextrose (BBL, Sparks, MD, USA) supplemented with 0.5 g of agar, 0.84 g of Na_2_CO_3_ and 0.5 g of L-cysteine・HCl・H_2_O (pH 7.2). Dilutions were then inoculated onto 3 non-selective and 8 selective agar media (Table 2). Beerens agar medium [6] was also included for isolation of bifidobacteria. The plating was not replicated in the present study. Bacteria were identified at the levels of genus or family based on colony form, Gram staining, cell morphology, and growth under aerobic conditions. Bacterial numbers were expressed as the log_10_ number of bacteria per gram wet weight of feces.

**Table 2:** The media and cultural method for comprehensive investigation of intestinal microbiota.

Medium	Organisms usually enumerated	Incubation method	Incubation days
Nonselective media			
EG agar	Anaerobes	Steel wool method	3
BL agar	Anaerobes	Steel wool method	3
Trypticase Soy Blood agar	Aerobes	Air	1
Selective media			
BS agar	Bifidobacteria	Steel wool method	3
Beerens agar	Bifidobacteria	Steel wool method	3
LBS agar (modified)	Lactobacilli	Steel wool method	3
ES agar	Eubacteria	Steel wool method	3
NBGT agar	Bacteroidaceae	Steel wool method	3
CW agar	Lecithinase-positive clostridia	Steel wool method	3
VS agar	*Veillonella* and *Megasphaera*	Steel wool method	3
DHL agar	Enterobacteriaceae	Air	1
TATAC agar	Enterococci	Air	2
PEES agar	Staphylococci	Air	2
Potato dextrose agar	Yeasts and molds	Air	2

One to three per sample bacterial colonies suggestive of bifidobacteria and/or lactobacilli by the shape of colony and cell morphology were isolated from modified LBS, BS, and Beerens agar and stored at -80°C for further identification.

### Species identification of isolates

The DNA of isolated bacteria was extracted using a Simple Prep^®^ DNA Extraction Kit (Takara Bio Inc., Kusatsu, Shiga), according to the manufacturer’s protocol. The 16S rRNA gene was amplified from the DNA extracts using a Bacterial 16S rDNA PCR Kit (Takara Bio), and the PCR products were purified with NucleoSpin^®^ Gel and PCR Clean-up (Macherey-Nagel GmbH & Co. KG., Düren, Germany), according to the manufacturer’s instructions. The sequences of the purified products were analyzed by FASMAC Co., Ltd. sequencing service (Kanagawa, Japan). The sequences obtained were compared with those available in nucleic acid databases using the EzTaxon-e database (http://eztaxon-e.ezbiocloud.net/) [7] and species were identified as the top hit species with <98% similarity using the EzTaxon-e database.

### Quantification of *Lactobacillus* group and *Bifidobacterium* by real-time PCR

Bacterial DNA was extracted directly from fecal samples using QIAamp DNA Stool Mini Kit (QIAGEN GmbH, Hilden, Germany), according to the manufacturer’s protocol. The real-time PCRs were carried out by using MyiQ real-time PCR system (Bio-Rad, Tokyo, Japan). The reaction mixture (20 μl) contained 10 μl of the iQ SYBER Green Supermix (Bio-Rad), 1 μl of fecal DNA, and 400 μmol/l of each primer. For quantification of *Lactobacillus* group, the primers 5’-AGCAGTAGGGAATCTTCCA-3’ (forward) and 5'-CACCGCTACACATGGAG-3' (reverse) were used [8]. For quantification of *Bifidobacterium*, the primers g-Bifid-F (5’-CTCCTGGAAACGGGTGG -3’) and g-Bifid-R (5’-GGTGTTCTTCCCGATATCTACA -3’) were used [9]. The thermal program consisted of initial denaturation at 95°C for 3 min followed by 40 cycles of at 94°C for 10 s, primer annealing at optimum temperature for 30 s and at 72°C for 40 s. The annealing temperatures for *Lactobacillus* group and *Bifidobacterium* were 58°C and 55°C, respectively. Fluorescent products were detected at the last step of each cycle. Melting curve analysis of the product was performed after completion of the amplifications to determine the specificity of the PCR. A plasmid containing a partial sequence of the 16S rRNA gene identical to the targeted bacteria was constructed in our laboratory and used as a standard DNA for the real-time PCR.

### Statistical analysis

Bacterial numbers and gene copy numbers of *Lactobacillus* group and *Bifidobacterium* were compared among 5 groups by Tukey’s t-test. Detection frequencies were compared by Fisher’s exact tests. All statistical analyses were performed using EZR (Saitama Medical Center, Jichi Medical University, Saitama, Japan), which is a graphical user interface for R (The R Foundation for Statistical Computing, Vienna, Austria). [10]

## Results

### The composition of the intestinal microbiota of cats in different age groups

The compositions of the fecal microbiota in different age groups are shown in Table 3. Enterococci were detected in almost all animals, while the mean number of enterococci was significantly lower in the senile group than that in the pre-weanling group. On the other hand, bifidobacteria were detected in only 1 out of 10 aged and 3 out of 10 senile cats. Lactobacilli were also detected only in 3 out of 10 pre-weanling, 4 out of 10 young, 1 out of 10 aged and 4 out of 10 senile cats. Although clostridia were detected in only 1 out of 10 aged cats, this bacterial group was detected in many of the cats of four other age groups.

**Table 3:** Fecal microbiota of the different age groups of cats.

Bacterial groups	Pre-weanling (n = 10)		Weanling (n = 10)		Young (n = 10)		Aged (n = 10)		Senile (n = 10)	
Bacteroidaceae	9.7 ± 0.6^a^	(10)	10.4 ± 0.4^a^^,^^b^^,^^c^	(10)	9.8 ± 0.2^b^	(10)	10.0 ± 0.3	(10)	9.7 ± 0.3^c^	(10)
Bifidobacteria		(0)		(0)		(0)	7.3	(1)	8.6 ± 0.9	(3)
Clostridia	8.0 ± 1.9	(7)^a^	7.3 ± 0.6^a^	(8)^b^	8.7 ± 0.6	(10)^c^	5.2	(1)^a^^,^^b^^,^^c^^,^^d^	8.9 ± 0.9^a^	(10)^d^
Eubacteria	8.3 ± 2.2^a^^,^^b^	(6)	10.5 ± 0.3^a^	(10)	9.6 ± 0.2^b^	(10)	9.5 ± 0.5	(10)	9.4 ± 0.4	(10)
*Megasphaera*		(0)^a^		(0)^b^	6.9 ± 1.5	(6)^a^^,^^b^^,^^c^		(0)^c^	5.9 ± 0.9	(2)
Lactobacilli	7.3 ± 2.2	(3)		(0)	4.4 ± 1.6	(4)	9.3	(1)	5.3 ± 3.2	(4)
Enterobacteriaceae	9.4 ± 0.4^a^^,^^b^^,^^c^^,^^d^	(10)	6.0 ± 1.2^a^	(10)	6.8 ± 0.7^b^	(10)	5.9 ± 2.2^c^	(10)	6.6 ± 1.3^d^	(10)
Enterococci	8.8 ± 0.9^a^	(10)	7.0 ± 1.2	(10)	7.1 ± 1.6	(10)	7.8 ± 2.1	(10)	6.1 ± 2.4^a^	(9)
Staphylococci		(0)^a^^,^^b^		(0)^c^^,^^d^	3.8 ± 0.9	(10)^a^^,^^c^^,^^e^		(0)^e^^,^^f^	4.3 ± 1.1	(6)^b^^,^^d^^,^^f^
Total count	10.1 ± 0.5^a^		10.8 ± 0.2^a^^,^^b^^,^^c^^,^^d^		10.1 ± 0.2^b^		10.2 ± 0.3^c^		10.1 ± 0.2^d^	

Mean ± SD of log_10_/g feces when the organism was present (number of subjects in which the organism was detected)

^a-f^The same superscript letters in the same horizontal line indicate significant differences (*P*<0.05)

The number of Enterobacteriaceae was significantly higher in the pre-weanling kittens compared with four other age groups. Compared with the pre-weanling, young and senile cats, the number of Bacteroidaceae was significantly higher in the weanling cats. The number of eubacteria was significantly higher in the pre-weanling kittens compared with the weanling and young cats. *Megasphaera* and staphylococci were detected only in the young and senile cats. The detection rate of *Megasphaera* in the young cats was higher compared with three other age groups, while those of staphylococci in the young and senile cats were also higher compared with the three other groups. The total bacterial count became the highest in the weanling cats.

### Species identification of lactobacilli and bifidobacteria

Since bifidobacteria and/or lactobacilli in the intestinal microbiota are thought to play beneficial roles in maintaining host well-being and often used as probiotics, isolated strains of bifidobacteria and lactobacilli were subjected to nucleotide sequencing of the 16S rRNA genes to delineate them at the species level (Table 4). Among the 4 strains of bifidobacteria, one strain from aged cat was identified as *Bifidobacterium longum* subsp. *longum*, while one *B*. *longum* subsp. *suis* and two *B*. *pseudocatenulatum* strains were isolated from senile cats.

**Table 4:** Occurrence of species of bifidobacteria and lactobacilli in feces of cats in the different age groups.

Species	Pre-weanling	Weanling	Young	Aged	Senile
*B*. *longum* subsp. *longum*				1	
*B*. *longum* subsp. *suis*					1
*B*. *pseudocatenulatum*					2
*L*. *animalis*				1	2
*L*. *fermentum*					1
*L*. *johnsonii*					1
*L*. *mucosae*					1
*L*. *reuteri*	5		3		

In pre-weanling and young groups, only *Lactobacillus reuteri* was detected, while this species was not detected from aged and senile cats. Various species other than *L*. *reuteri*, *L*. *johnsonii*, *L*. *animalis*, *L*. *fermentum* and *L*. *mucosae*, were isolated from the senile group (Table 4).

### The numbers of gene copies of *Lactobacillus* group and *Bifidobacterium*

The numbers of 16S rRNA gene copies of *Lactobacillus* group and *Bifidobacterium* were measured by real-time PCR as an indicator of cell number. With PCR method, *Lactobacillus* group was detected from all samples. There was no significant difference in detection rate and number of gene copies of *Lactobacillus* group among age groups. On the other hand, *Bifidobacterium* were detected from 35 out of 50 samples and the number of gene copies of *Bifidobacterium* in senile cats was significantly higher than that in young and aged cats (Table 5).

**Table 5:** The relative abundance of *Lactobacillus* group and *Bifidobacterium*.

Bacterial groups	Pre-weanling (n = 10)		Weanling (n = 10)		Young (n = 10)		Aged (n = 10)		Senile (n = 10)	
*Lactobacillus* group	7.0 ± 0.7	(10)	5.9 ± 0.3	(10)	6.2 ± 0.2	(10)	6.2 ± 1.2	(10)	6.5 ± 1.0	(10)
*Bifidobacterium*	5.8 ± 0.5	(4)	5.1 ± 0.8	(6)	4.8 ± 0.6^a^	(9)	4.8 ± 0.3^b^	(8)	5.8 ± 0.8^a^^,^^b^	(8)

Mean ± SD of log_10_/g feces when the organism was present (number of subjects in which the organism was detected by real-time PCR)

^a,b^The same superscript letters in the same horizontal line indicate significant differences (*P*<0.05)

## Discussion

In this study, we analyzed the composition of the intestinal microbiota of cats in different age groups. Although we did not follow up the same cohort of animals for prolonged years, our results seem to suggest aging of the intestinal microbiota in cats.

Although the population of eubacteria was lower in the pre-weanling cats compared with the weanling and young cats, Bacteroidaceae and eubacteria were predominant throughout life. Enterobacteriaceae and enterococci were predominant in the pre-weanling kittens, while the number of Enterobacteriaceae and enterococci appeared to decrease as the animals got older. Lactobacilli were detected from less than half of the animals in all age groups, and bifidobacteria were detected only from one aged and three senile cats. These results basically agree with the previous findings that the detection rate of lactobacilli was low and bifidobacteria was hardly detected from all age groups in cats [4].

The present study employed a culture-based method, which was essentially the same as those described by Mitsuoka et al. [1, 5], to analyze the composition of the intestinal microbiota of cats, because we aimed to not only identify beneficial age-related changes in the composition of feline intestinal microbiota but also to isolate particular bacteria for future development of probiotics targeting cats. On the other hand, recent studies in which they employed a culture independent method, pyrosequencing, have reported that lactobacilli and bifidobacteria are abundant in cats [11]. Therefore, we further tried to quantify these two bacterial groups using molecular method, real-time PCR. Unlike culture-based method, *Lactobacillus* group were detected from all cats and *Bifidobacterium* was detected from 70% of cats by real-time PCR. The selective media used in the present study might not be perfectly suitable for lactobacilli and bifidobacteria of cat origin. Furthermore, molecular method, containing real-time PCR, is able to not only live but also dead bacterial DNA. However, the highest gene copy numbers detected were 7.0 ± 0.7 (log_10_/g wet weight of feces) in pre-weanling group for *Lactobacillus* group and 5.8 ± 0.8 in pre-weanling and senile group for *Bifidobacterium*. The present study revealed that lactobacilli and bifidobacteria are the minor components of cat intestinal microbiota.

Recent advances in the molecular methods for the studies on microbiota have made quick and more comprehensive analysis of the intestinal microbiota possible. A previous study utilizing molecular method reported that the composition of the intestinal microbiota of four healthy conventionally raised cats (ages 13–18 months) were comprised of about 68% of phyla Firmicutes group, followed by Proteobacteria (14%), Bacteroidetes (10%), Fusobacteria (5%) and Actinobacteria (4%) [12]. Another previous study with Illumina sequencing has reported that *Lactobacillus* and *Bifidobacterium* are extremely common (35% and 20% respectively) in cats at about 18 weeks of age [11]. Furthermore, the previous study with pyrosequencing reported that the most abundant phylum was Firmicutes, followed by Actinobacteria in the intestinal microbiota of twelve healthy cats (ages 0.7–6.7 years) [13]. Most of the bacteria classified in lactobacilli, enterococci, clostridia and eubacteria in this study belong to phylum Firmicutes, while most of the bacteria classified in Bacteroidaceae in this study belong to the phyla Bacteroidetes or Fusobacteria. In addition, bacteria identified as Enterobacteriaceae in this study belongs to phylum Proteobacteria, indicating that our results obtained by the culture method did not coincide with these two previous findings obtained by using molecular techniques. On the other hands, the previous study with metagenomics analysis reported that the composition of intestinal microbiota of five healthy cats (from 3–16 years) were comprised of ~68% of phylum Bacteroides/Chlorobi group, followed by Firmicutes (~13%) and Proteobacteria (~6%) [14]. From the perspective of that phylum Bacteroides/Chlorobi group was the most dominant, this study corresponds to our results obtained by using the culture method. To elucidate the cause of these contradictions, the influence of various factors including age, breed and environment such as diet and rearing conditions should be further studied.

It has been reported that the composition of the intestinal microbiota of human begins changes with age. The dominance of bifidobacteria observed in infancy is not evident in the middle-aged population, and there are slight reductions in total bacterial counts. Furthermore, bifidobacteria become completely undetectable in some individuals in old age, while the prevalence and the numbers of *Clostridium perfringens*, *Lactobacillus*, Enterobacteriaceae and *Enterococcus* markedly increase [1, 4]. Although bifidobacteria, the most predominant bacteria in infants and one of the predominant bacteria in adults in humans [4], are thought to confer some health benefits, the finding in this study that bifidobacteria were isolated only from one aged and three senile cats suggested that bifidobacteria may not play important roles in cats in contrast to humans. Furthermore, although our previous study showed that lactobacilli may exert some health benefits in dogs similar to those anticipated for bifidobacteria in humans [2], lactobacilli were only minor constituents in cats. On the other hand, enterococci were predominant among lactic acid producing bacteria in pre-weanling cats, and the number of enterococci appeared to decrease in elder individuals, in contrast to the finding in humans that the numbers of enterococci in human gut increased with age [4]. It seems, therefore, likely that enterococci may have some health benefits in cats similar to those anticipated for bifidobacteria in human and lactobacilli in dogs. The roles played by each component bacteria in microbiota might be different depending on the species of animals in question.

Although the number of *Lactobacillus* strains isolated by culture-based method was limited, *L*. *reuteri* was the only species isolated from pre-weanling and young cats, while various species of *Lactobacillus* other than *L*. *reuteri* were isolated from elder cats. In dogs, *L*. *johnsonii* strains were mostly isolated from pre-weanling dogs, while *L*. *animalis* was isolated from all age groups except for the senile group [2], suggesting that *L*. *johnsonii* might be a specific species for infant dogs. The present study clearly indicated that the age related changes in the intestinal microbiota are different between cats and dogs, also at the level of bacterial species.

The present study showed that the intestinal microbiota of cats undergoes age-dependent changes at the levels of both bacterial groups and species, as in the case of human and dog intestinal microbiota, although the changes in senile cats should be further studied because only the senile cats were kept under different conditions. The present study also suggested that the roles played by some intestinal bacterial groups of cats might be different from those of humans and dogs. Our results suggested that *Bifidobacterium* and *Lactobacillus* species may not be as important for cat health as in the case of humans and dogs. Instead, enterococci were suggested to be one of the major lactic acid producing bacteria playing important roles to control intestinal conditions of cats. The present study indicates the importance of development of probiotics specific to cats. Considering that some major human probiotic species are predominant in healthy infants and decrease in elder individuals [15–17], further studies on the age related changes of enterococci at the species level should be performed. It is known that age-associated changes, such as reduced digestive function and constipation, often occur in senile cats and these symptoms may also be related to intestinal microbiota in cats [18]. It is expected that bacterial species specific to cats will play an important part in reducing these symptoms as probiotics.

## References

[1] Mitsuoka T. Establishment of intestinal bacteriology. Biosci Microbiota Food Health. 2014; 33: 99–116. doi: 10.12938/bmfh.33.99 25032084 10.12938/bmfh.33.99 PMC4096684

[2] Masuoka H, Shimada K, Kiyosue-Yasuda T, Kiyosue M, Oishi Y, Kimura S, et al Transition of the intestinal microbiota of dogs with age. Biosci Microbiota Food Health. 2017; 36: 27–31. doi: 10.12938/bmfh.BMFH-2016-021 28243548 10.12938/bmfh.BMFH-2016-021 PMC5301054

[3] Smith HW. The development of the flora of the alimentary tract in young animals. J Pathol Bacteriol. 1965; 90: 495–513. 4285022

[4] Mitsuoka T, Kaneuchi C. Ecology of the bifidobacteria. Am J Clin Nutr. 1977; 30: 1799–1810. 920640 10.1093/ajcn/30.11.1799

[5] Hirayama K, Itoh K, Takahashi E, Mitsuoka T. Comparison of composition of faecal microbiota and metabolism of faecal bacteria among `human-flora-associated' mice inoculated with faeces from six different human donors. Microb Ecol Health Dis. 1995; 8: 199–211.

[6] Beerens H. An elective and selective isolation medium for *Bifidobacterium* spp. Lett Appl Microbiol. 1990; 11: 155–157.

[7] Kim OS, Cho YJ, Lee K, Yoon SH, Kim M, Na H, et al Introducing EzTaxon-e: a prokaryotic 16S rRNA gene sequence database with phylotypes that represent uncultured species. Int J Syst Evol Microbiol. 2012; 62: 716–721. doi: 10.1099/ijs.0.038075-0 22140171 10.1099/ijs.0.038075-0

[8] Rintilä T, Kassinen A, Malinen E, Krogius L, Palva A. Development of an extensive set of 16S rRNA-targeted primers for quantification of pathogenic and indigenous bacteria in faecal samples by real-time PCR. J Appl Microbiol. 2004; 97: 1166–1177. doi: 10.1111/j.1365-2672.2004.02409.x 15546407 10.1111/j.1365-2672.2004.02409.x

[9] Matsuki T, Watanabe K, Fujimoto J, Takada T, Tanaka R. Use of 16S rRNA gene-targeted group-specific primers for real-time PCR analysis of predominant bacteria in human feces. Appl Environ Microbiol. 2004; 70: 7220–7228. doi: 10.1128/AEM.70.12.7220-7228.2004 15574920 10.1128/AEM.70.12.7220-7228.2004 PMC535136

[10] Kanda Y. Investigation of the freely available easy-to-use software ‘EZR’ for medical statistics. Bone Marrow Transplant. 2013; 48: 452–458. doi: 10.1038/bmt.2012.244 23208313 10.1038/bmt.2012.244 PMC3590441

[11] Deusch O, O’Flynn C, Colyer A, Swanson KS, Allaway D, Morris P. A longitudinal study of the feline faecal microbiome identifies changes into early adulthood irrespective of sexual development. PLOS ONE. 2015; 10: e0144881 doi: 10.1371/journal.pone.0144881 26659594 10.1371/journal.pone.0144881 PMC4682054

[12] Ritchie LE, Steiner JM, Suchodolski JS. Assessment of microbial diversity along the feline intestinal tract using 16S rRNA gene analysis. FEMS Microbiol Ecol. 2008; 66: 590–598. doi: 10.1111/j.1574-6941.2008.00609.x 19049654 10.1111/j.1574-6941.2008.00609.x

[13] Garcia-Mazcorro JF, Lanerie DJ, Dowd SE, Paddock CG, Grützner N, Steiner J M. Effect of a multi-species synbiotic formulation on fecal bacterial microbiota of healthy cats and dogs as evaluated by pyrosequencing. FEMS Microbiol Ecol. 2011; 78: 542–554. doi: 10.1111/j.1574-6941.2011.01185.x 22067056 10.1111/j.1574-6941.2011.01185.x

[14] Tun HM, Brar MS, Khin N, Jun L, Hui R KH, Dowd SE, et al Gene-centric metagenomics analysis of feline intestinal microbiome using 454 junior pyrosequencing. J Microbiol Methods. 2012; 88: 369–376. doi: 10.1016/j.mimet.2012.01.001 22265636 10.1016/j.mimet.2012.01.001 PMC7114314

[15] Mitsuoka T. History and evolution of probiotics. Jpn J Lactic Acid Bact. 2011; 22: 26–37.

[16] Maldonado J, Canabate F, Sempere L, Vela F, Sanchez AR, Narbona E. Human milk probiotic *Lactobacillus fermentum* CECT5716 reduces the incidence of gastrointestinal and upper respiratory tract infections in infants. J Pediatr Gastroenterol Nutr. 2012; 54: 55–61. doi: 10.1097/MPG.0b013e3182333f18 21873895 10.1097/MPG.0b013e3182333f18

[17] Menard O, Butel MJ, Gaboriau-Routhiau V, Waligora-Dupriet AJ. Gnotobiotic mouse immune response induced by *Bifidobacterium* sp. strains isolated from infants. Appl Environ Microbiol. 2008; 74: 660–666. doi: 10.1128/AEM.01261-07 18083875 10.1128/AEM.01261-07 PMC2227707

[18] Barrette D. Alimentation du chien et du chat ages. Can Vet J. 1992; 33: 349–350. 17424011 PMC1481245

